# Promotion of Interface Fusion of Solid Polymer Electrolyte and Cathode by Ultrasonic Vibration

**DOI:** 10.3390/s22051814

**Published:** 2022-02-25

**Authors:** Hui Wang, Haoran Ke, Yizhe Chen, Jinhuo Wang, Fei Yan, Xiaodong Cui

**Affiliations:** 1 Hubei Key Laboratory of Advanced Technology for Automotive Components, Wuhan University of Technology, Wuhan 430070, China; huiwang@whut.edu.cn (H.W.); 250592@whut.edu.cn (H.K.); 2 Hubei Collaborative Innovation Center for Automotive Components Technology, Wuhan 430070, China; 823416242@whut.edu.cn; 3 Fujian Key Laboratory of Functional Materials and Applications, Xiamen University of Technology, Xiamen 361024, China; wangjinhuo@hotmail.co.uk; 4 Hubei Research Center for New Energy & Intelligent Connected Vehicle, Wuhan University of Technology, Wuhan 430070, China; fyan001@whut.edu.cn

**Keywords:** solid polymer electrolyte, ultrasonic fusion, interface resistance, wireless sensors

## Abstract

All-solid-state polymer lithium batteries have good safety, stability, and high energy densities and are employed in wireless sensors. However, the solid contact between the polymer electrolyte and the cathode leads to high interface resistance, limiting the broad application of solid-state lithium batteries. This paper proposes an ultrasonic fusion method to reduce the interface resistance between the polymer electrolyte and the cathode. The method applied a high-frequency ultrasonic vibration technique to impact the polymer electrolyte/cathode structure, melting the electrolyte at the interface and thus generating good contact at the interface. The experimental results showed that the ultrasonic fusion method decreased the interface resistance between the polymer electrolyte and the cathode by 96.2%. During the ultrasonic fusion process, high-frequency ultrasonic vibrations generated high temperatures at the interface, and the polymer electrolyte became molten, improving the contact between the electrolyte and the cathode. The ultrasonic fusion method eliminated the gaps at the interface, and the interface became more compact. Furthermore, ultrasonic vibrations made the molten electrolyte fill the holes in the cathode, and the contact area was enhanced, providing more Li^+^ ions transmission paths.

## 1. Introduction

Wireless Sensor Networks (WSNs) have attracted a significant amount of attention thanks to their pervasive nature and wide range of applications in emerging fields, such as the Internet of Things and Cyber–Physical Systems [1,2]. Since sensor nodes are located in hard-to-reach locations, it is usually expensive and inconvenient to replace the nodes’ batteries. The use of sensor networks is severely restricted by the energy constraints posed by sensor nodes [3,4]. All-solid-state polymer lithium batteries are employed as power sources for wireless sensors because of their excellent safety, stability, and high energy density [5,6]. However, the high interface resistance between the solid electrolyte and the cathode severely limits the broad application of all-solid-state polymer lithium batteries.

The contact at the interface between the polymer electrolyte and the cathode is poor because of their solid properties, and the existence of tiny gaps at the interface significantly reduces the contact area. Lithium ions cannot pass through the gaps, resulting in high interface resistance [7]. In order to improve the contact at the interface and eliminate the gaps to reduce the interface resistance between the solid electrolyte and the cathode, researchers have conducted much research in recent years. Zipei Wan et al. [8] used polyethylene oxide (PEO) and lithium bis(trifluoromethylsulfonyl)imide (LiTFSI) as the cathode binders and prepared a composite electrolyte matrix embedded with Li_7_La_3_Zr_2_O_12_ (LLZO) nanowires. The PEO in the cathode and the composite electrolyte were fused at a high temperature to form an integrated electrolyte/electrode structure, which effectively enhanced the interface compatibility and stability between the electrolyte and cathode and ensured efficient lithium-ion transport at the interface. Kyusung Park et al. [9] intensely studied the cathode/electrolyte interface between LiCoO_2_ and LLZO. LiBO_3_, an inorganic material with ion conductivity and a low melting point, was used as a binder and inserted into the interface between the electrolyte and the electrode. After heating, the contact between the electrode material and the electrolyte was effectively improved. Jingang Zheng et al. [10] introduced a double ionic–electronic transfer interface layer at the interface between the electrolyte and electrode by polymerizing 2,2′-bithiophene in polyethylene oxide electrolyte. The formation of the conductive polythiophene layer with superior interfacial stability and contact properties at the interface resulted in a sevenfold reduction in interface resistance. Zhuo Li and Xin Guo [11] reduced the interface resistance between the composite solid electrolyte and the cathode by creating an integrated interfacial structure. Polyethylene oxide, lithium bis(trifluoromethylsulfonyl)imide, and ionic liquids were used to form a viscoelastic interface. As a result, an interface between the solid electrolyte and the cathode with tight contact, low interface resistance, and fast ion transport was achieved. Zelin Yang et al. [12] used an interface adhesion strategy to reduce the interface resistance between the solid electrolyte and the cathode. A thin layer of adhesive PEO was used as an interfacial binder to connect the dense Li_1.3_Al_0.3_Ti_1.7_(PO_4_)_3_ ceramic electrolyte to the solid electrode. Additionally, the interface was enhanced by using a PEO adhesive instead of a poly(1,1-difluoroethylene) (PVDF) adhesive to bond the cathode assembly. The results showed that the interface resistance between the solid electrolyte and the electrode was reduced by two orders of magnitude.

In recent years, the ultrasonic vibration-assisted processing method has become a hot research topic. This processing method is applied to bond thermoplastic composites, and its mechanism involves the frictional heating of molecules using low-amplitude, high-frequency ultrasonic vibration [13,14]. Irene Fernandez Villegas and Regis van Moorleghem [15] demonstrated that high-frequency ultrasonic vibrations at tens of thousands of times per second could produce partial high temperatures that caused thermoplastics to melt. When two pieces of plastic were placed together, the interface could melt, and the materials could be fused. Yibo Sun et al. [16] established an experimental platform to study vibration transmission and investigated the interface fusion process of thermoplastic polymers under ultrasonic vibration. The results showed that the mobility of the polymer was further enhanced with the effect of ultrasonic vibration. The experimental results indicated that the ultrasonic vibration promoted the formation of the fused interface and reduced the generation of defects. Habibi et al. [17] investigated the effect of ultrasonic vibration on the wetting of the poly(3,4-ethylenedioxythiophene): poly(styrene sulfonic acid) (PEDOT: PSS) films used in solar cells. The results showed that ultrasonic vibration improved surface wetting by promoting droplet spreading and repairing the dewetted areas. Weibing Guo et al. [18] used the molecular dynamics (MD) simulation method to analyze the effect of ultrasonic vibration on the wetting of Pb droplets on the Al (1 0 0) surface. It was found that the interaction between Pb and Al was small in the absence of ultrasonic vibration. Under ultrasonic vibration, aluminum atoms dissolved in the Pb droplets, and Pb atoms diffused into the aluminum lattice, producing violent surface alloying.

To solve the problem of poor compatibility between the solid electrolyte and the cathode, an ultrasonic fusion method is proposed in this paper to promote the contact between the solid electrolyte and the cathode and reduce the interface resistance. Firstly, the polymer electrolytes and LiFePO_4_ cathodes were prepared, and ultrasonic vibration was applied to promote the fusion of the solid electrolytes and the cathodes. Then, the ultrasonic vibration parameters were optimized in orthogonal experiments, and the effect of the ultrasonic fusion method on reducing the interface resistance was analyzed and verified. Finally, the mechanism of the ultrasonic fusion method on reducing the interface resistance was studied.

## 2. Materials and Methods

### 2.1. Materials

The matrix material used for the solid polymer electrolyte (SPE) is PEO (Mw = 10^6^ g/mol, Shanghai Kaiyuan Chemical Technology, Shanghai, China). The lithium salt is lithium bis(trifluoromethane)sulfonimide (LiTFSI, 99%, Shanghai Aichun Biological Technology, Shanghai, China). The solvents are acetonitrile (ACN, 99.8%, Hefei Jiankun Chemical, Hefei, China) and N-Methylpyrrolidone (NMP, 99.5%, Wuxi Yatai United Chemical, Wuxi, China). LiFePO_4_ (Shenzhen Kejing Zhida Technology, Shenzhen, China) and SUPER-P (Shenzhen Kejing Zhida Technology, Shenzhen, China) are used for the active cathode material and conductive agent. The binder is poly(1,1-difluoroethylene) (PVDF, 99.8%, Sinopharm Chemical Reagent, Shanghai, China). The cathode current collector is aluminum foil (Taizhou Yajun Battery Material, Taizhou, China).

### 2.2. Experimental Method

#### 2.2.1. Preparation of Solid Polymer Electrolyte

The PEO-based solid polymer electrolyte was prepared using the solution casting method. Before preparation, PEO and LiTFSI were dried in a vacuum drying oven at 60 °C for 24 h to remove residual moisture. In an argon-filled glove box (O_2_ < 0.01 ppm, H_2_O < 0.01 ppm), 1.25 g PEO and 0.45 g LiTFSI (EO:Li = 18:1) were weighed and slowly added into 30 mL acetonitrile in turn, and the solution was magnetically stirred for 24 h until PEO and LiTFSI were wholly dissolved. The electrolyte solution was poured into a polytetrafluoroethylene (PTFE) mold. Then, the mold was placed horizontally in the glove box for 6 h to allow the acetonitrile solvent to evaporate naturally. After that, it was dried in a vacuum drying oven at 50 °C for 5 h to remove the residual acetonitrile solvent from the electrolyte. Finally, the electrolyte films were slowly removed from the PTFE mold and measured with a digital micrometer. The prepared electrolyte had a uniform thickness of about 100 μm. The electrolyte films were cut into disks with a diameter of 16 mm (the size of the diaphragm for a button cell) using a slicer and placed in the glove box.

#### 2.2.2. Preparation of LiFePO_4_ Cathode

The cathode is made up of active materials, conductive agents, a binder, and current collectors. The active material used for the cathode was LiFePO_4_, the conductive agent was SUPER-P, the binder was PVDF, and the current collector was aluminum foil. Here, 0.15 g PVDF was weighed and added into 1.35 g NMP solvent, and the solution was magnetically stirred until the PVDF was completely dissolved. A binder solution with a mass fraction of 10% was obtained. Then, 0.8 g LiFePO_4_ and 0.1 g SUPER-P were weighed and added to a mortar, and the mixture was ground for 1 h. After that, 1 g of the binder solution was added to the mixture and ground for 1 h to obtain a uniformly mixed slurry. A 100 μm doctor blade was used to coat the slurry on aluminum foil evenly. The cathodes were dried in a vacuum drying oven at 60 °C for 6 h to allow the solvent to evaporate, and were then vacuum dried at 120 °C for 4 h to remove residual solvent. A manual microtome was applied to cut the cathode into disks with a diameter of 13 mm and placed in the glove box for later use.

#### 2.2.3. Ultrasonic Fusion Method

The traditional method used pressure to combine the solid electrolyte and the cathode, resulting in poor contact at the solid–solid interface. In this study, the ultrasonic fusion method applied ultrasonic vibration to impact the electrolyte/cathode structure to form a good contact interface between the solid electrolyte and the cathode. The impact between the solid electrolyte and the cathode generated a high enough temperature to melt the electrolyte. Thus, the cathode was wetted by molten electrolyte, and the solid–solid interface was substituted with the solid–liquid interface during the formation. Furthermore, the impact promoted the penetration of the molten electrolyte into the cathode. The contact area was enhanced between the solid electrolyte and the cathode. Therefore, a good contact interface was achieved.

The equipment used to fuse the solid electrolyte and the cathode was the Taiwan MAXWIND^®^ ME-1800 ultrasonic vibration platform. The ultrasonic vibration platform consisted of an ultrasonic generator, a transducer, an amplitude transformer, and a sonotrode. When the ultrasonic equipment works, the generator converts the low-frequency electric signal into a high-frequency electric signal matched with the transducer. Then, the transducer converts the high-frequency electric signal into ultrasonic vibration. The amplitude of the generated ultrasonic vibration is so minimal that it is not large enough to achieve the fusion of the solid electrolyte and the cathode. Therefore, the amplitude transformer is applied to increase the amplitude of the ultrasonic vibration. Finally, the ultrasonic vibration is transmitted to the position to be fused through the sonotrode. In addition, the ultrasonic vibration platform can pressurize the cylinder using an air pump, and the ultrasonic pressure can be changed by adjusting the pressure in the cylinder.

**Figure 1:**
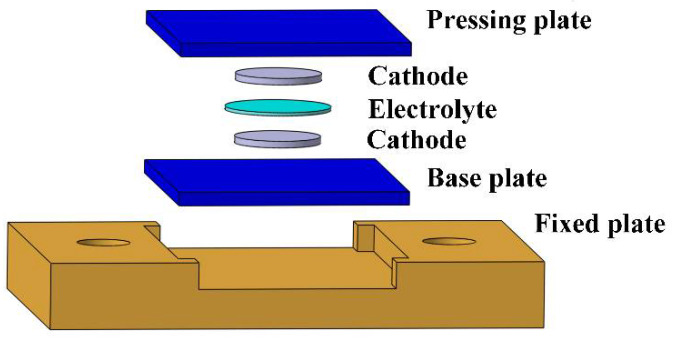
The ultrasonic fusion mold.

An ultrasonic fusion mold was applied to prevent the solid electrolyte and the cathode from being damaged during the high-frequency vibration, as shown in Figure 1. Both the pressing plate and the base plate were carbon fiber-reinforced plastic (CFRP) laminates, which could effectively transmit the ultrasonic vibration and avoid rigid contact. The fixed plate was made of a 7075 aluminum alloy and was bolted to the experimental platform to prevent horizontal movement during ultrasonic fusion from tearing the solid electrolyte and the cathode.

The cathode symmetric batteries, with the cathode on both sides of the electrolyte, were applied to study the interface resistance between the solid electrolyte and the cathode. Firstly, the electrolyte and the cathode were put in the ultrasonic fusion mold, as shown in Figure 1. Next, the ultrasonic frequency was set to 20 kHz. A pulse mode was applied to avoid damaging the electrolytes by overheating them. In one cycle on this mode, the ultrasonic vibration operated for 4 s and was suspended for 4 s. The ultrasonic time (ultrasonic vibration operating time) was 4~16 s, the ultrasonic amplitude was 14~17 μm, and the ultrasonic pressure was 0.08~0.32 MPa. The processing parameters were optimized via an orthogonal experiment, conducted as follows. The sonotrode was pushed down to touch the pressing plate, and the ultrasonic generator was turned on. The ultrasonic vibration was applied to the interface between the electrolyte and the cathode. Finally, the sonotrode was pushed up, and the fused cathode symmetric battery was removed from the mold. The cathode symmetric batteries made using the traditional method were recorded as the Reference Group, and those with the ultrasonic fusion method were recorded as the Ultrasonic Group. In the traditional method, the cathode symmetric battery was assembled with a pressure of 5 MPa.

### 2.3. Orthogonal Experimental Design

The orthogonal experiment method was applied to obtain the optimal processing parameters for the reduction of the interface resistance between the solid electrolyte and the cathode. The main processing parameters include the ultrasonic time, ultrasonic amplitude, and ultrasonic pressure. Based on preliminary trials, we observed the influence of the parameters on the sample. The orthogonal experiment was designed using Minitab (V17.1) software, and the orthogonal L16 (4^3^) table was obtained, as shown in Table 1. The orthogonal experiment was carried out according to the table. In order to ensure the validity of the data, the experiments were repeated five times, and the mean values were recorded.

### 2.4. Characterization

#### 2.4.1. Direct Current (DC) Polarization

In order to prevent the battery from short circuiting, one of the basic requirements for solid electrolytes is that the electrolyte cannot conduct electrons. The ability of PEO-based solid polymer electrolyte films to conduct electrons can be analyzed with the DC polarization test. The SS (stainless steel)/SPE/SS structure was tested using the Shanghai Chenhua CHI760E electrochemical workstation at room temperature. The polarization voltage was set at 3 V, and the test time was 300 min.

#### 2.4.2. Alternating Current (AC) Impedance

AC impedance spectroscopy of the cathode symmetric battery was measured by placing the prepared electrolyte film between two LiFePO_4_ cathodes. The test instrument was the Shanghai Chenhua CHI760E electrochemical workstation, the test frequency range was from 0.1 Hz to 1 MHz, and the signal amplitude was 5 mV.

#### 2.4.3. Linear Sweep Voltammetry (LSV)

The electrochemical window is an essential indicator of the stability of solid electrolytes. The electrochemical window of the SS/SPE/Li structure was examined by linear sweep voltammetry. The test instrument was the Shanghai Chenhua CHI760E electrochemical workstation. The sweep voltage was increased from open circuit potential to 6 V at a rate of 0.5 mV/s.

#### 2.4.4. Scanning Electron Microscope (SEM)

The microstructure of the interface between the electrolyte and the cathode was observed using the TESCAN MIRA4 field emission scanning electron microscope. Before the observation, the electrolyte/cathode structure was fractured in liquid nitrogen. After gold coating, the fracture surface was observed.

#### 2.4.5. Fourier Transform Infrared (FTIR) Spectroscopy

The change of functional groups at the interface was observed using the Nicolet 6700 Fourier Transform Infrared Spectrometer produced by Thermo Fisher Scientific. Powder was achieved by scraping at the interface, and this was used to prepare the KBr pellet to perform the infrared scanning test. The wave number was from 400 cm^−1^ to 4000 cm^−1^.

## 3. Results and Discussion

### 3.1. Interface Resistance

#### 3.1.1. AC Impedance

**Figure 2:**
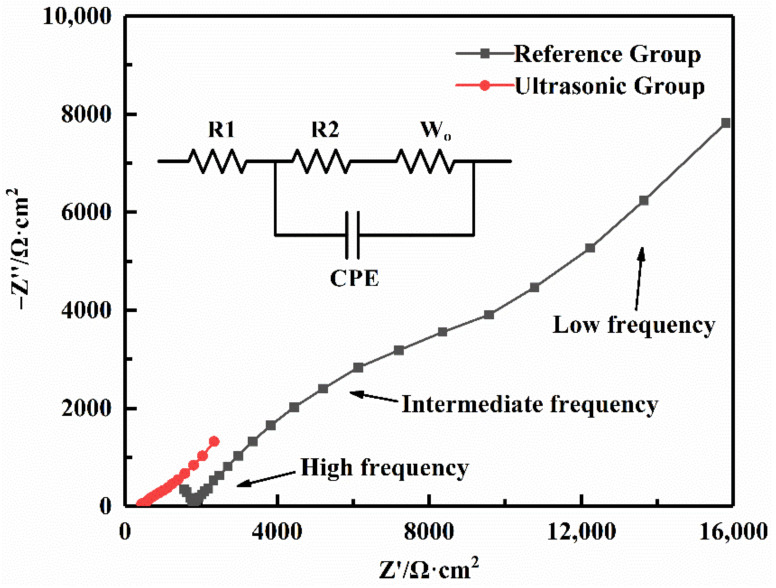
AC impedance spectroscopy of the cathode symmetric batteries.

Figure 2 shows the AC impedance spectrum of the cathode symmetric batteries of the Reference Group and the Ultrasonic Group at a test temperature of 30 °C. For the Ultrasonic Group, scheme 7 in Table 1 was selected as an example (the other schemes are similar). The ultrasonic time was 8 s, the ultrasonic amplitude was 16 μm, and the ultrasonic pressure was 0.32 MPa. There was a straight line in the low-frequency region, which reflected the solid-state diffusion process of lithium ions in the polymer electrolyte. The intermediate frequency region was a circular arc, which was related to the interface resistance between the electrolyte and the cathode. The intersection of the high-frequency region and the horizontal axis reflected the electrolyte resistance. In order to understand the AC impedance curve more clearly, ZView (Version 3.1) software was applied to fit the measured impedance data to the fitting circuit shown in the illustration of Figure 2. In the fitting circuit, R1 is the electrolyte resistance, R2 is the symmetric battery interface resistance (since the test structure is a cathode symmetric battery, its value is twice the interface resistance between the electrolyte and LiFePO_4_ cathode), W_o_ is the diffusion impedance, and CPE is the double-layer capacitance at the electrolyte/cathode interface. After fitting (as shown in Appendix A), only the electrolyte resistance and interface resistance are shown in Table 2.

As shown in Table 2, for the cathode symmetric batteries made via the traditional method, the interface resistance was much higher than the electrolyte resistance. It indicated that the interface resistance was the main part of the internal resistance of the solid battery. Therefore, it is essential to reduce the interface resistance between the electrolyte and the cathode. The ultrasonic fusion method reduced both the electrolyte resistance and the interface resistance. The electrolyte resistance was reduced from 1784.9 Ω·cm^2^ to 630.8 Ω·cm^2^. This was because the effect of ultrasonic vibration decreased the crystallinity of PEO and promoted the conduction of lithium ions in the solid electrolyte [19]. Furthermore, the interface resistance was reduced from 7739.9 Ω·cm^2^ to 528.5 Ω·cm^2^, and it was decreased by 93.2%. The results demonstrated that the ultrasonic fusion method could effectively reduce the interface resistance between the polymer electrolyte and the LiFePO_4_ cathode.

#### 3.1.2. Main Effect Analysis

**Figure 3:**
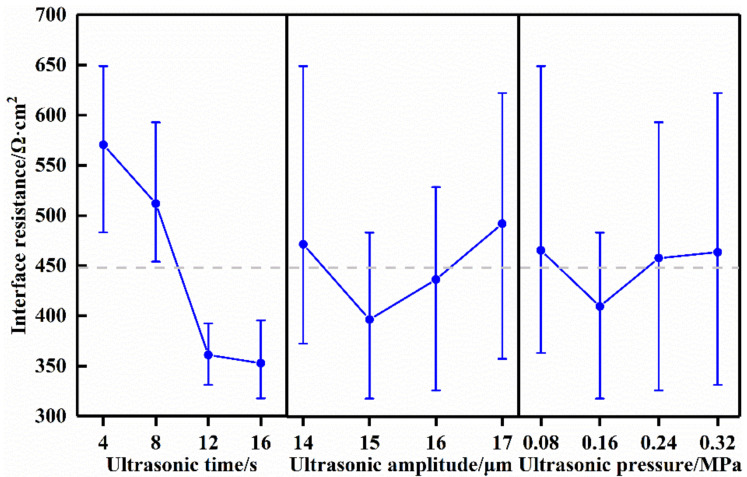
Main effects plot: the effect of each factor on the interface resistance.

The results of the orthogonal experiment are listed in Table 1. From Table 1, all the interface resistance scores of the cathode symmetric batteries made by the ultrasonic fusion method were lower than those for the traditional method. This demonstrated that the ultrasonic fusion method had a significant effect on reducing interface resistance. In order to investigate the effect of the processing parameters on interface resistance and to achieve minimum interface resistance, the “Analysis of Taguchi Design” in Minitab was used to analyze the orthogonal experiment data. The output mean value response is shown in Table 3, and the main effect plot is shown in Figure 3.

The response of the mean values reflected the influence of each factor on reducing the interface resistance between the polymer electrolyte and LiFePO_4_ cathode. The main effects plot was used to check the difference between the level means. From Table 3, the ultrasonic fusion method with different process factors had different effects on the interface resistance. The mean response results showed that the influence of each processing factor descends in the order of ultrasonic time > ultrasonic amplitude > ultrasonic pressure. Therefore, the ultrasonic time had the most significant effect on the interface resistance, while the ultrasonic pressure had the least significant effect. From Figure 3, the ultrasonic fusion method with processing factors of different levels achieved different interface resistance values. The three factors are independent of each other. If the interface resistance was minimum for a factor, the corresponding level was optimal. When the ultrasonic time was 16 s, a minimum interface resistance was obtained. For ultrasonic amplitude and ultrasonic pressure, these values were 15 μm and 0.16 MPa, respectively. Therefore, the ultrasonic time, ultrasonic amplitude, and ultrasonic pressure should take the values of 16 s, 15 μm, and 0.16 MPa, respectively, which are the optimal processing parameters of the ultrasonic fusion method.

#### 3.1.3. Verification

From Section 3.1.2, when the ultrasonic time, the ultrasonic amplitude, and the ultrasonic pressure were 16 s, 15 μm, and 0.16 MPa, respectively, the lowest interface resistance was achieved. Because the scheme was not included in the orthogonal experiments in Table 1, it should be verified through additional experiments. The interface resistance results of the Ultrasonic Group with the optimal processing parameters and the Reference Group at different test temperatures are listed in Table 4.

As shown in Table 4, with the temperature increasing, the interface resistance between the solid electrolyte and the LiFePO_4_ cathode decreased. At 30 °C, the Reference Group had a high interface resistance due to poor physical contact between the solid electrolyte and the cathode. The ultrasonic fusion method reduced the interface resistance from 7739.9 Ω·cm^2^ to 293.6 Ω·cm^2^ at 30 °C, and it was decreased by 96.2%. At 50 °C, the solid polymer electrolyte became soft, and the contact between the electrolyte and the cathode improved. Thus, the interface resistance at that temperature was significantly lower than that at 30 °C. The ultrasonic fusion method reduced the interface resistance from 502.1 Ω·cm^2^ to 33.25 Ω·cm^2^ at 50 °C, and it was decreased by 95.4%. At 70 °C, the temperature exceeded the melting point (60 °C) of PEO, and the polymer electrolyte became molten. The contact between the electrolyte and the cathode was further improved, and the interface resistance was significantly reduced. At this temperature, the interface resistance of the cathode symmetric batteries made by the ultrasonic fusion method was only 20.0 Ω·cm^2^, decreasing by 68.4%. Therefore, the ultrasonic fusion method could significantly reduce the interface resistance between the solid electrolyte and the LiFePO_4_ cathode. 

### 3.2. Polymer Electrolyte Performance

#### 3.2.1. DC Polarization

**Figure 4:**
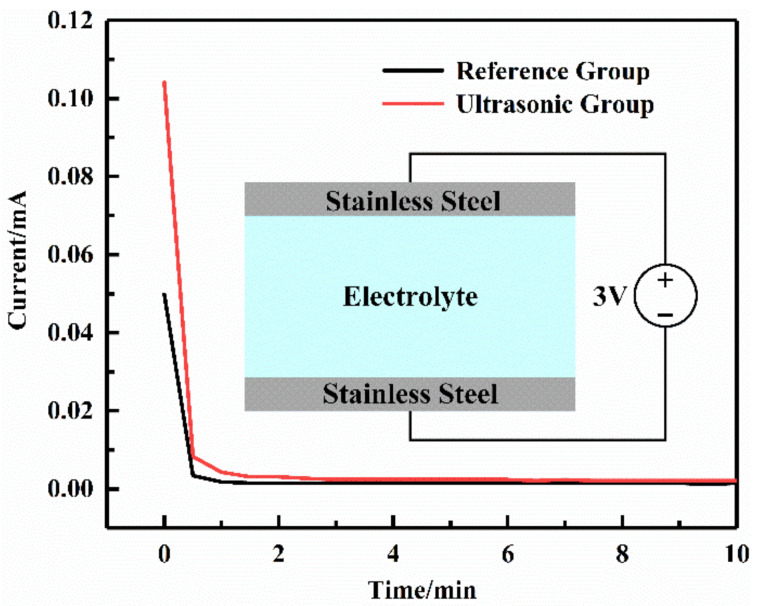
DC polarization test curves of solid polymer electrolyte.

In order to test the electronic insulation of the prepared polymer electrolyte, a DC polarization test was carried out. For the Ultrasonic Group, the optimal processing parameters were used. As shown in Figure 4, the illustration was a schematic diagram of the test structure, with stainless steel blocking electrodes on both sides of the electrolyte. At the beginning of the test, the ions in the polymer electrolyte migrated directionally at a polarization voltage of 3 V to generate a polarization current. As the test progressed, the current rapidly decreased from a high initial value to about zero and remained stable. All ions in the electrolyte had migrated completely, and thus no current was generated. The weak steady current shown at the bottom of the curve resulted from the electrolyte conducting the electrons. The stable currents of the Reference Group and the Ultrasonic Group were 1.2 μA and 0.7 μA, respectively. The ultrasonic fusion method increased the stable current. However, it was still so small that the electrons could not be effectively conducted in the electrolytes, which would not result in a short circuit. Therefore, the prepared polymer electrolyte had good electronic insulation, and the ultrasonic fusion method would not damage it.

#### 3.2.2. Electrochemical Window

**Figure 5:**
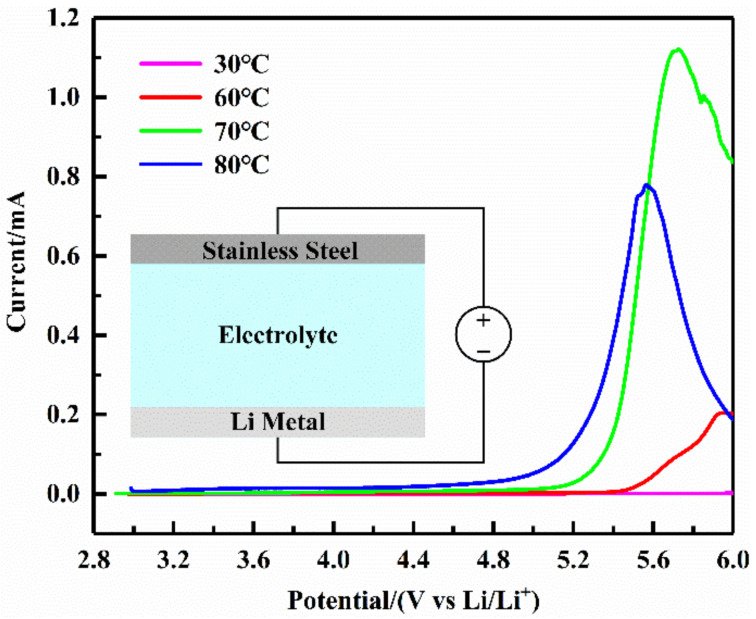
The linear sweep voltammetry curves of the Ultrasonic Group at different temperatures.

It was necessary for polymer electrolytes to have an excellent electrochemical stability window. A linear sweep voltammetry test was carried out to check the electrochemical stability of the polymer electrolyte of the Ultrasonic Group. As shown in Figure 5, at low potentials, the current kept stable and remained about zero. As the potential increased, the PEO in the polymer electrolyte began to oxidize and decompose, generating a large current. Before the current peak, the amount of oxidatively decomposed PEO increased as the potential rose, and thus the current increased. After the current peak, the amount of PEO participating in the oxidation decomposition at the interface decreased as the reaction proceeded, and thus the current decreased. At 30 °C, the voltage increased to 6 V (vs. Li/Li^+^), and no current was generated, indicating that the electrochemical stability window of the polymer electrolyte at this temperature reached more than 6 V. As the temperature increased, the decomposition potential of the electrolyte decreased. However, even at 80 °C, the stable electrochemical window of the electrolyte could reach 4.6 V (vs. Li/Li^+^). Therefore, the polymer electrolyte of the Ultrasonic Group had an excellent electrochemical stability window.

### 3.3. Mechanism Analysis

#### 3.3.1. Thermal Effect

**Figure 6:**
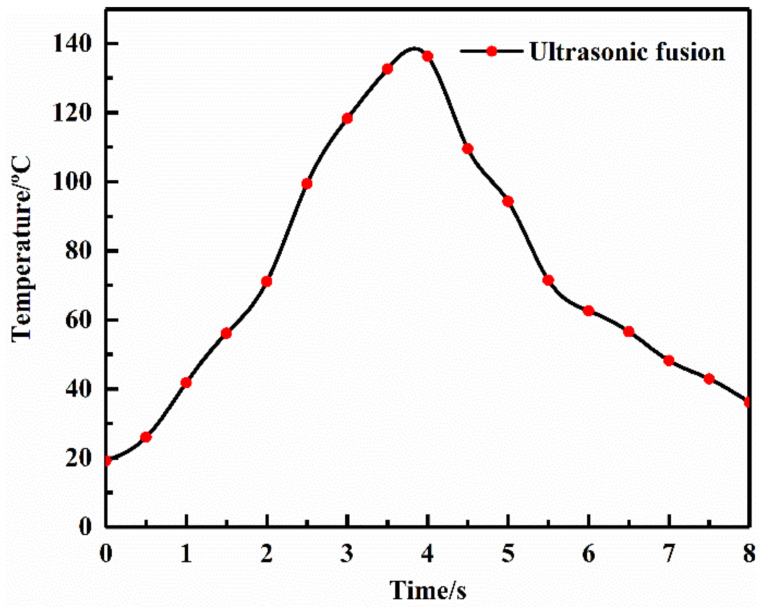
Interface temperature during ultrasonic fusion.

The high-frequency ultrasonic vibration resulted in interface impact tens of thousands of times per second, which could generate high temperatures at the interface [15,20]. A thermocouple thermometer EL-R19 was used to monitor the interface temperature during the ultrasonic fusion. The results are shown in Figure 6, where the temperature in one pulse cycle was presented. The temperature raised rapidly with ultrasonic vibration operating, reaching 60 °C within 2 s. After the ultrasonic vibration was suspended, the temperature dropped. The average temperature was about 75 °C in one cycle. This demonstrated that the ultrasonic fusion process would cause the temperature to rise at the interface. The melting point of PEO is 60 °C, and thus the PEO is in a molten state during the ultrasonic fusion process. In order to examine the thermal effect on the interface resistance, other polymer electrolyte/LiFePO_4_ cathode structures were heated to 75 °C and pressed with a pressure of 0.16 MPa. The temperature was similar with average temperature in the ultrasonic fusion process, and the pressure was the same, but ultrasonic vibration was not applied. Those were recorded as the Hot-pressed Group. The Hot-pressed Group and the Reference Group, formed with the traditional method, were subjected to AC impedance tests at different temperatures to check their interface resistance. The test results are listed in Table 5.

Table 5 shows that there was a significant difference in interface resistance between the Reference Group and the Hot-pressed Group. At 30 °C, the interface resistance of the Reference Group was 7739.9 Ω·cm^2^, while that of the Hot-pressed Group was only 1316.7 Ω·cm^2^. The hot press process decreased the interface resistance between the solid electrolyte and LiFePO_4_ cathode at that temperature. At 50 ℃, the interface resistance was decreased from 502.1 Ω·cm^2^ to 143.6 Ω·cm^2^. At 70 °C, the interface resistance of the Hot-pressed Group was 27.9 Ω·cm^2^, which was still lower than that of the Reference Group. It demonstrated that heating could effectively reduce the interface resistance. The thermal effect played a role in the ultrasonic fusion process and contributed to reducing the interface resistance. However, by comparing the interface resistance of the Hot-pressed Group with that of the Ultrasonic Group in Table 4, it can be seen that there was still a large difference in the interface resistance between the Hot-pressed Group and the Ultrasonic Group. Therefore, the thermal effect of the ultrasonic fusion method reduced the interface resistance, but it was not the only reason for the ultrasonic fusion method to reduce the interface resistance.

#### 3.3.2. Mechanical Effect

**Figure 7:**
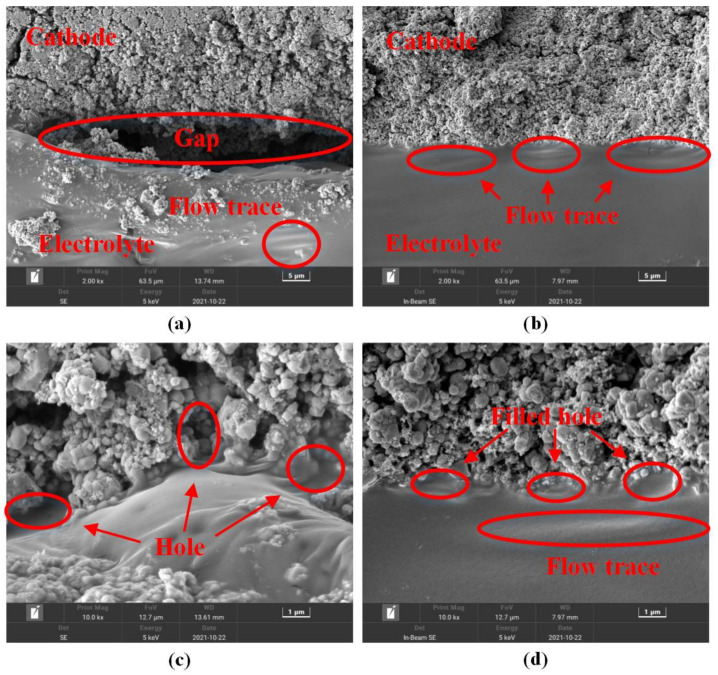
Electrolyte/cathode interface morphologies from: (**a**) Hot-pressed Group, 2000×; (**b**) Ultrasonic Group, 2000×; (**c**) Hot-pressed Group, 10,000×; (**d**) Ultrasonic Group, 10,000×.

The ultrasonic vibration, as a kind of mechanical wave, can make the polymer behave in a pattern of forced vibration and, thus, exerts alternative stress onto it [21]. In order to study the mechanical effect of ultrasonic vibration on reducing the interface resistance, the SEM test was carried out to observe the morphology of the interface between the electrolyte and the LiFePO_4_ cathode. The interface morphologies of the Hot-pressed Group and the Ultrasonic Group are shown in Figure 7. The polymer electrolyte/LiFePO_4_ cathode structures of the Hot-pressed Group were pressed with a pressure of 0.16 MPa at 75 ℃. For the Ultrasonic Group, the ultrasonic time, the ultrasonic amplitude, and the ultrasonic pressure were 16 s, 15 μm, and 0.16 MPa, respectively.

Figure 7a,c show the morphology of the interface formed by the hot-pressed process, while Figure 7b,d show that by the ultrasonic fusion method. In Figure 7a, one can see that gaps existed at the interface between the electrolyte and LiFePO_4_ cathode, which would result in high interface resistance. However, for the cathode symmetric battery made using the ultrasonic fusion method, no obvious gaps were observed at the interface, and the electrolyte and the LiFePO_4_ cathode were in good contact, as shown in Figure 7b,d. In Figure 7a,b, flow traces of the polymer electrolyte can be observed. When the polymer electrolyte was in the molten state, the electrolyte was compressed using the hot press or ultrasonic process, and the flow of electrolyte was produced. For the Hot-pressed Group, the flow trace was inside the electrolyte. This was because the hot press process caused the compression of the whole structure and mainly produced the internal molten electrolyte flow, which had little influence on the interface. However, the ultrasonic fusion method produced an impact at the interface, making the molten electrolyte diffuse at the interface. From Figure 7c,d, it can be seen that the polymer electrolyte wetted the material in the cathode. This was because the polymer electrolyte was heated to become molten by the hot press or ultrasonic process, and the fluidity was improved. For the Hot-pressed Group, the holes existed in the cathode, and the electrolyte and the cathode were in poor contact. However, the ultrasonic fusion method caused the electrolyte to penetrate into the LiFePO_4_ cathode and filled the holes in the cathode. This indicated that the ultrasonic fusion method improved the penetration capacity of the molten polymer electrolyte, which was beneficial to forming a tight interface with high adhesion. The morphology of the interface formed by the ultrasonic fusion method was indistinct. Therefore, the polymer electrolyte was in good contact with the cathode, which increased the contact area between the electrolyte and the cathode and promoted the reduction of the interface resistance.

**Figure 8:**
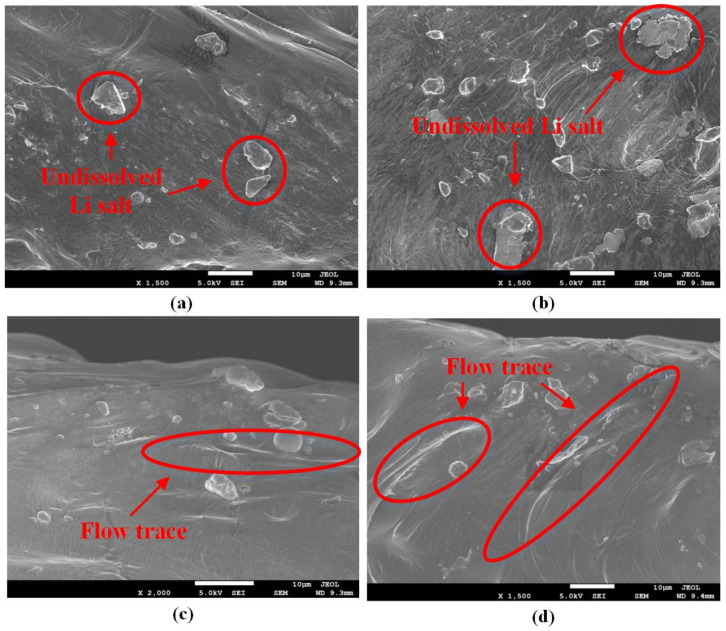
Surface morphologies of the electrolyte from: (**a**) prepared; (**b**) Reference Group; (**c**) Hot-pressed Group; (**d**) Ultrasonic Group.

In order to investigate the influence of different methods on the surface of the electrolytes, different methods were applied to solid polymer electrolytes only. The SEM test was carried out to observe the surface morphologies of the electrolytes. Figure 8 shows the surface morphologies of the electrolytes from different groups. In Figure 8, one can see that all the polymer electrolytes contained undissolved lithium salt, which was precipitated out from the electrolytes. Flow traces were observed in both the electrolytes of the Hot-pressed Group and the Ultrasonic Group. For the Reference Group, no flow trace was observed on the surface of the electrolyte. This was because the pressure applied to the solid polymer electrolyte was not able to make the electrolyte flow. The surface of the electrolyte of the Ultrasonic Group had more flow traces than that of the Hot-pressed Group. This indicated that the electrolyte of the Ultrasonic Group had better fluidity than that of the Hot-pressed Group.

#### 3.3.3. FTIR Analysis

**Figure 9:**
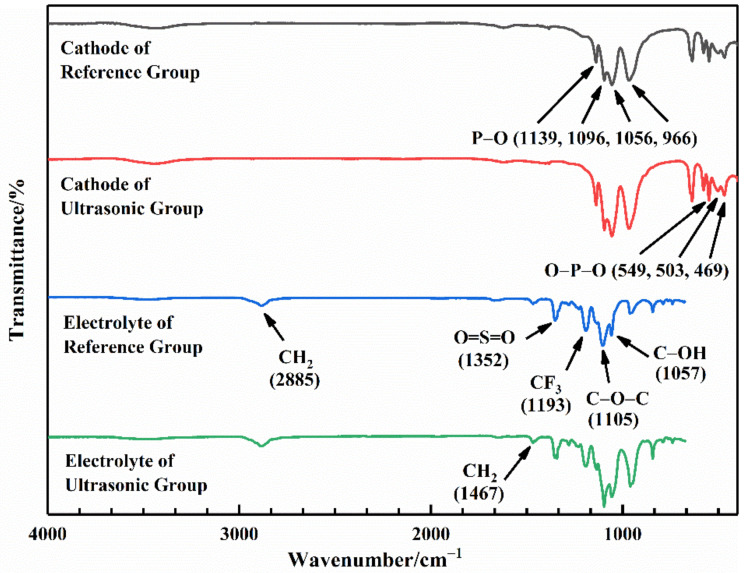
FTIR test results of the electrolyte and the cathode.

The FTIR spectroscopies of the electrolyte and the cathode at the interface of the Reference Group and the Ultrasonic Group are shown in Figure 9. The symmetric stretching vibrations of P–O were found at 966 cm^−1^ and 1139 cm^−1^, and the intramolecular antisymmetric stretching vibrations of P–O were observed at 1056 cm^−1^ and 1096 cm^−1^. The sharp peaks at 469 cm^−1^ and 503 cm^−1^ corresponded to the symmetric bending vibrations of O–P–O, and the peak at 549 cm^−1^ was due to the antisymmetric bending vibrations of O–P–O. The peaks corresponding to LiFePO_4_ were observed both in the cathode of the Reference Group and the Ultrasonic Group. The FTIR test results of the cathode showed that the ultrasonic fusion method would not change the chemical composition of the cathode. For the electrolyte, the peaks at 2884 cm^−1^ and 1467 cm^−1^ were attributed to the stretching and bending vibrations of CH_2_ in the PEO chains. The peak at 1105 cm^−1^ was due to the C–O–C in the PEO, which had lone electron pairs and could coordinate with Li^+^ ions to transport Li^+^ ions. The C–OH in the PEO was found at 1057 cm^−1^, which was the end of the PEO chain. These peaks were the characteristic peaks of the PEO. Furthermore, the stretching vibration of the O=S=O was observed at 1352 cm^−1^, and the stretching vibration of the CF_3_ was identified at 1193 cm^−1^. The two peaks corresponded to LiTFSI in the electrolyte. Both the PEO and LiTFSI were identified in the FTIR spectroscopy of the electrolyte, but no obvious difference in the peaks was found between the two groups. It demonstrated that the ultrasonic fusion method would not alter the chemical composition of the electrolyte and the cathode at the interface. Therefore, the ultrasonic fusion method had no adverse effect on the chemical composition of the polymer electrolyte and the LiFePO_4_ cathode.

## 4. Conclusions

This paper proposed an ultrasonic fusion method to reduce the interface resistance between the polymer electrolyte and LiFePO_4_ cathode. The method applied high-frequency ultrasonic vibration to generate impact and melt the solid electrolyte to form a well-contacted interface between the electrolyte and the cathode. The orthogonal experiment was used to study the effects of three processing parameters on reducing the interface resistance and to determine the optimal parameters of the ultrasonic fusion method. The thermal effect of the ultrasonic fusion method on reducing the interface resistance was examined. The interface morphologies were investigated to analyze the mechanical effect of the ultrasonic fusion method. Furthermore, an FTIR analysis was carried out to check the chemical composition of the electrolyte and the cathode at the interface. The main conclusions are as follows:

1. The proposed ultrasonic fusion method can significantly reduce the interface resistance between the polymer electrolyte and the cathode without adversely affecting the electronic insulation and electrochemical stability of the polymer electrolyte. The ultrasonic fusion method with the optimal processing parameters decreased the interface resistance by 96.2%.
2. The order of the influence of the three processing parameters on the interface resistance was ultrasonic time > ultrasonic amplitude > ultrasonic pressure.
3. The ultrasonic fusion method caused the temperature to rise at the interface. The thermal effect in the ultrasonic fusion process contributed to reducing the interface resistance. However, it was not the only reason why the ultrasonic fusion method reduced the interface resistance.
4. The ultrasonic fusion method produced an impact at the interface, redistributing the molten electrolyte at the interface and improving the penetration capacity of the molten polymer electrolyte. In addition, the ultrasonic fusion method would not change the chemical composition of the electrolyte and the cathode at the interface.

In this paper, the ultrasonic fusion method was first proposed, which could significantly reduce the interface resistance between the electrolyte and the cathode without introducing extra materials.

## Figures and Tables

**Table 1:** Orthogonal schemes and experimental results.

Scheme	Ultrasonic Time (s)	Ultrasonic Amplitude (μm)	Ultrasonic Pressure (MPa)	Interface Resistance (Ω·cm^2^)
1	4	14	0.08	648.$9_{-16.9}^{+13.4}$
2	4	15	0.16	483.$2_{-26.3}^{+19.8}$
3	4	16	0.24	527.$8_{-12.1}^{+12.9}$
4	4	17	0.32	622.$1_{-33.0}^{+20.6}$
5	8	14	0.16	472.$0_{-20.1}^{+28.8}$
6	8	15	0.08	454.$1_{-18.7}^{+15.2}$
7	8	16	0.32	528.$5_{-20.0}^{+22.6}$
8	8	17	0.24	593.$0_{-44.2}^{+47.8}$
9	12	14	0.24	392.$4_{-10.9}^{+7.1}$
10	12	15	0.32	331.$4_{-9.4}^{+14.6}$
11	12	16	0.08	363.$2_{-13.9}^{+20.8}$
12	12	17	0.16	357.$4_{-6.8}^{+9.7}$
13	16	14	0.32	372.$5_{-22.5}^{+18.7}$
14	16	15	0.24	317.$8_{-7.5}^{+14.7}$
15	16	16	0.16	325.$9_{-10.3}^{+11.4}$
16	16	17	0.08	395.$4_{-19.2}^{+25.0}$

**Table 2:** Electrolyte resistance and interface resistance of the cathode symmetric batteries.

	Electrolyte Resistance (Ω·cm^2^)	Interface Resistance (Ω·cm^2^)
Reference Group	1784.9	7739.9
Ultrasonic Group	630.8	528.5

**Table 3:** Mean value response (unit: Ω·cm^2^).

Level	Factor		
	Ultrasonic Time (s)	Ultrasonic Amplitude (μm)	Ultrasonic Pressure (MPa)
1	570.5	471.5	465.4
2	511.9	396.6	409.6
3	361.1	436.4	457.7
4	352.9	492.0	463.6
Delta	217.6	95.4	55.8
Row rank	1	2	3

**Table 4:** Interface resistance of the Reference Group and the Ultrasonic Group at different temperatures (Ω·cm^2^).

	30 °C	50 °C	70 °C
Reference Group	7739.9	502.1	63.2
Optimal Group	293.6	33.25	20.0

**Table 5:** Interface resistance of the Hot-pressed Group and the Reference Group at different temperatures (Ω·cm^2^).

	30 °C	50 °C	70 °C
Reference Group	7739.9	502.1	63.2
Hot-pressed Group	1316.7	143.6	27.9

## Data Availability

Not applicable.

## Appendix A

Tien Quang Nguyen (*Journal of The Electrochemical Society*, 2018, 165, E826, doi: 10.1149/2.1151814jes) studied alternating current impedance spectroscopy similar to the process demonstrated in Figure 2 and used a fitting circuit similar to that in Figure 2 to fit it. The alternating current impedance spectroscopy in Figure 2 had a positive Z’ shift relative to that in the reference. Thus, another resistor was connected in series in the fitting circuit. Woosung Choi (*Journal of Electrochemical Science and Technology*, 2020, 11(1), 1–13, doi: 10.33961/jecst.2019.00528) and Evan L. Anderson (*Analytical Chemistry*, 2016, 88(19), 9738–9745, doi: 10.1021/acs.analchem.6b02641) also used a fitting circuit to fit the alternating current impedance spectroscopy similarly to Figure 2. Parameters of the fit of the Reference Group and the Ultrasonic Group are shown in Table A1 and Table A2. R1 is the electrolyte resistance, and R2 is the symmetric battery interface resistance. The CPE is defined by two values, CPE-T and CPE-P. The Wo is described by the parameters of Wo-R, Wo-T, and Wo-P. Values for the “Sum of Squares” of the Reference Group and the Ultrasonic Group are shown in the tables. The values are below 0.01, which also justified the fitting circuit. Not all the fitting results are listed in Table 2. The aim of this article was to reduce the interface resistance. Thus, only electrolyte resistance (R1) and interface resistance (a half of R2) are listed in Table 2.

**Table A1:** Parameters of the fit of the Reference Group.

Parameters	Value
R1 (Ω·cm^2^)	1784.9
R2 (Ω·cm^2^)	15,479.8
W_o_-R (Ω·cm^2^)	23,673
W_o_-T (s)	85.95
W_o_-P (-)	0.65467
CPE-T (Ω^−1^·cm^−2^·s^p^)	0.00011033
CPE-P (-)	0.49537
Sum of Squares (-)	0.0062221

**Table A2:** Parameters of the fit of the Ultrasonic Group.

Parameters	Value
R1 (Ω·cm^2^)	630.8
R2 (Ω·cm^2^)	1057
W_o_-R (Ω·cm^2^)	4429
W_o_-T (s)	67.64
W_o_-P (-)	0.45553
CPE-T (Ω^−1^·cm^−2^·s^p^)	0.00041719
CPE-P (-)	0.58429
Sum of Squares (-)	0.0014421

## References

[1] Shaikh F.K., Zeadally S. (2016). Energy harvesting in wireless sensor networks: A comprehensive review. Renew. Sustain. Energy Rev..

[2] Kandris D., Nakas C., Vomvas D., Koulouras G. (2020). Applications of wireless sensor networks: An up-to-date survey. Appl. Syst. Innov..

[3] Tuna G., Gungor V. (2016). Energy harvesting and battery technologies for powering wireless sensor networks. Industrial Wireless Sensor Networks.

[4] Prauzek M., Konecny J., Borova M., Janosova K., Hlavica J., Musilek P. (2018). Energy harvesting sources, storage devices and system topologies for environmental wireless sensor networks: A review. Sensors.

[5] Danilov D., Niessen R., Notten P. (2010). Modeling all-solid-state Li-ion batteries. J. Electrochem. Soc..

[6] Manthiram A., Yu X., Wang S. (2017). Lithium battery chemistries enabled by solid-state electrolytes. Nat. Rev. Mater..

[7] Luo W., Gong Y., Zhu Y., Li Y., Yao Y., Zhang Y., Fu K., Pastel G., Lin C.F., Mo Y. (2017). Reducing interfacial resistance between garnet-structured solid-state electrolyte and Li-metal anode by a germanium layer. Adv. Mater..

[8] Wan Z., Lei D., Yang W., Liu C., Shi K., Hao X., Shen L., Lv W., Li B., Yang Q.H. (2019). Low resistance–integrated all-solid-state battery achieved by Li_7_La_3_Zr_2_O_12_ nanowire upgrading polyethylene oxide (PEO) composite electrolyte and PEO cathode binder. Adv. Funct. Mater..

[9] Park K., Yu B.-C., Jung J.-W., Li Y., Zhou W., Gao H., Son S., Goodenough J.B. (2016). Electrochemical nature of the cathode interface for a solid-state lithium-ion battery: Interface between LiCoO_2_ and garnet-Li_7_La_3_Zr_2_O_12_. Chem. Mater..

[10] Zheng J., Sun C., Wang Z., Liu S., An B., Sun Z., Li F. (2021). Double ionic-electronic transfer interface layers for all solid-state lithium batteries. Angew. Chem. Int. Ed..

[11] Li Z., Guo X. (2021). Integrated interface between composite electrolyte and cathode with low resistance enables ultra-long cycle-lifetime in solid-state lithium-metal batteries. Sci. China Chem..

[12] Yang Z., Yuan H., Zhou C., Wu Y., Tang W., Sang S., Liu H. (2020). Facile interfacial adhesion enabled LATP-based solid-state lithium metal battery. Chem. Eng. J..

[13] Gouin O’Shaughnessey P., Dubé M., Fernandez Villegas I. (2016). Modeling and experimental investigation of induction welding of thermoplastic composites and comparison with other welding processes. J. Compos. Mater..

[14] Ning F., Cong W. (2020). Ultrasonic vibration-assisted (UV-A) manufacturing processes: State of the art and future perspectives. J. Manuf. Processes.

[15] Villegas I.F., van Moorleghem R. (2018). Ultrasonic welding of carbon/epoxy and carbon/PEEK composites through a PEI thermoplastic coupling layer. Compos. Part A Appl. Sci. Manuf..

[16] Sun Y., Wang F., Li F., Yang X. (2018). Study on vibration transmission and interfacial fusion in ultrasonic bonding process for thermoplastic micro joint. Adv. Polym. Technol..

[17] Habibi M., Eslamian M., Soltani-Kordshuli F., Zabihi F. (2016). Controlled wetting/dewetting through substrate vibration-assisted spray coating (SVASC). J. Coat. Technol. Res..

[18] Guo W., Ma K., Wang Q., Xue H. (2020). The wetting of Pb droplet on the solid Al surface can be promoted by ultrasonic vibration–Molecular dynamics simulation. Mater. Lett..

[19] Wang H., Cui X., Zhang C., Gao H., Du W., Chen Y. (2020). Promotion of Ionic Conductivity of PEO-Based Solid Electrolyte Using Ultrasonic Vibration. Polymers.

[20] Liu B., Xia H., Fei G., Li G., Fan W. (2013). High-Intensity Focused Ultrasound-Induced Thermal Effect for Solid Polymer Materials. Macromol. Chem. Phys..

[21] Peng K., Shahab S., Mirzaeifar R. (2020). Interaction of high-intensity focused ultrasound with polymers at the atomistic scale. Nanotechnology.

